# Patient Interactions With an Automated Conversational Agent Delivering Pretest Genetics Education: Descriptive Study

**DOI:** 10.2196/29447

**Published:** 2021-11-18

**Authors:** Daniel Chavez-Yenter, Kadyn E Kimball, Wendy Kohlmann, Rachelle Lorenz Chambers, Richard L Bradshaw, Whitney F Espinel, Michael Flynn, Amanda Gammon, Eric Goldberg, Kelsi J Hagerty, Rachel Hess, Cecilia Kessler, Rachel Monahan, Danielle Temares, Katie Tobik, Devin M Mann, Kensaku Kawamoto, Guilherme Del Fiol, Saundra S Buys, Ophira Ginsburg, Kimberly A Kaphingst

**Affiliations:** 1 Department of Communication University of Utah Salt Lake City, UT United States; 2 Cancer Control and Population Sciences Huntsman Cancer Institute Salt Lake City, UT United States; 3 Huntsman Cancer Institute University of Utah Salt Lake City, UT United States; 4 Perlmutter Cancer Center New York University Langone Health New York, NY United States; 5 Department of Biomedical Informatics University of Utah Salt Lake City, UT United States; 6 University of Utah Health Salt Lake City, UT United States; 7 Department of Medicine New York University Grossman School of Medicine New York University New York, NY United States; 8 Department of Population Health Sciences University of Utah Salt Lake City, UT United States; 9 Department of Population Health New York University Grossman School of Medicine New York University New York, NY United States; 10 Department of Internal Medicine University of Utah Salt Lake City, UT United States

**Keywords:** cancer, genetic testing, virtual conversational agent, user interaction, smartphone, mobile phone

## Abstract

**Background:**

Cancer genetic testing to assess an individual’s cancer risk and to enable genomics-informed cancer treatment has grown exponentially in the past decade. Because of this continued growth and a shortage of health care workers, there is a need for automated strategies that provide high-quality genetics services to patients to reduce the clinical demand for genetics providers. Conversational agents have shown promise in managing mental health, pain, and other chronic conditions and are increasingly being used in cancer genetic services. However, research on how patients interact with these agents to satisfy their information needs is limited.

**Objective:**

Our primary aim is to assess user interactions with a conversational agent for pretest genetics education.

**Methods:**

We conducted a feasibility study of user interactions with a conversational agent who delivers pretest genetics education to primary care patients without cancer who are eligible for cancer genetic evaluation. The conversational agent provided scripted content similar to that delivered in a pretest genetic counseling visit for cancer genetic testing. Outside of a core set of information delivered to all patients, users were able to navigate within the chat to request additional content in their areas of interest. An artificial intelligence–based preprogrammed library was also established to allow users to ask open-ended questions to the conversational agent. Transcripts of the interactions were recorded. Here, we describe the information selected, time spent to complete the chat, and use of the open-ended question feature. Descriptive statistics were used for quantitative measures, and thematic analyses were used for qualitative responses.

**Results:**

We invited 103 patients to participate, of which 88.3% (91/103) were offered access to the conversational agent, 39% (36/91) started the chat, and 32% (30/91) completed the chat. Most users who completed the chat indicated that they wanted to continue with genetic testing (21/30, 70%), few were unsure (9/30, 30%), and no patient declined to move forward with testing. Those who decided to test spent an average of 10 (SD 2.57) minutes on the chat, selected an average of 1.87 (SD 1.2) additional pieces of information, and generally did not ask open-ended questions. Those who were unsure spent 4 more minutes on average (mean 14.1, SD 7.41; *P*=.03) on the chat, selected an average of 3.67 (SD 2.9) additional pieces of information, and asked at least one open-ended question.

**Conclusions:**

The pretest chat provided enough information for most patients to decide on cancer genetic testing, as indicated by the small number of open-ended questions. A subset of participants were still unsure about receiving genetic testing and may require additional education or interpersonal support before making a testing decision. Conversational agents have the potential to become a scalable alternative for pretest genetics education, reducing the clinical demand on genetics providers.

## Introduction

### Background

Cancer genetic testing and the use of genomic information are central to the future of precision cancer medicine [1,2]. Effective communication of germline genetic results and family history of cancer to patients is key to their understanding of their own (and in many cases their family members’) cancer risks, evidence-based options for decision-making (ie, for cancer risk management), and in the case of individuals with cancer, their overall cancer trajectory, including treatment options [3]. Automated approaches to communication have begun to emerge as a way of meeting the expanding volume of testing in the context of a limited number of genetic counselors able to provide services [4]. One such approach is the use of an automated conversational agent to deliver cancer genetic services to supplement, or in lieu of, a genetic counselor. Conversational agents are automated, scripted, and responsive agents used to mimic human interactions. These agents use natural language processing to analyze user inputs and respond appropriately using human language via auditory or textual methods [4]. Conversational agents are increasingly popular in various health contexts, as they can be easily accessed through smartphones, tablets, laptops, or desktop computers. The agents are fairly accessible to most adults in the United States, of whom 75% report having at least one smartphone [5]. With an exponential growth in genetic testing as a way to identify individuals with inherited cancer susceptibility, conversational agents present an innovative approach to broadening access to clinical cancer genetics services in the face of limited health professionals with genomics expertise while encouraging wider use of cancer genetic testing incorporated in health care settings [6,7].

The delivery of health services through conversational agents in research contexts has been successfully tested in various health domains, such as mental health, asthma, diabetes management, and physical activity uptake [8]. Conversational agents have been found to help health care providers lower the rates of depression and anxiety [9-11] and also improve adherence to treatment for asthma, diabetes, and pain [12-14]. Recent research has begun to use the conversational agent model of care to facilitate informed decision-making and technology use self-efficacy related to prostate cancer [15,16]. Other conversational agents in noncancer settings, specifically mental health and lifestyle change interventions, identified in the Bibault et al [15] review, were found to be able to improve decision-making processes. Owens et al [16] developed iDecide, an embodied conversational agent–led, computer-based prostate cancer screening decision aid. Their findings showed that conversational agents were able to improve prostate cancer knowledge and informed decision-making self-efficacy and technology use self-efficacy among their target audience of African American men. Prior research on conversational agents has shown that most users are receptive to the use of this technology in health, although concerns related to accuracy, security, and lack of empathy have been raised [17,18]. Although conversational agents are a promising technology in various health contexts, more research is needed to examine their efficacy and implementation effectiveness and outcomes in genetic service delivery [19].

Conversational agents can be used in various ways in the context of cancer genetic services, especially in the context of hereditary cancers, for uses such as collecting initial data and communicating risk. Hereditary factors can affect the risk for many common adult-onset cancers (ie, breast, ovarian, colorectal, pancreas, and prostate) [20-26]. Cancer genetic testing generates quantifiable cancer risks, which can be helpful in directing the clinical management of patients. Such genetic risk assessments involve the collection of detailed patient information, such as their family’s cancer history; delivery of pretest genetic counseling to inform decisions about testing; and returning the results after testing. Only recently have clinical cancer care settings begun to leverage this technology to support patient information management in service delivery [4]. Conversational agents have been used in some of these processes, such as collecting patient data, providing genetic information, delivering results, and facilitating *cascade testing* of at-risk relatives in clinical settings [18,27-30].

There has been limited research investigating how users interact with conversational agents in various contexts, including health, education, and customer service [31,32]. For this study, interactions will be characterized as users’ reciprocal actions with our automated conversational agent. More research is needed on how conversational agents are used in health contexts, such as cancer genetic services and user interactions. For example, the Geisinger health care system has integrated a personalized conversational agent into the delivery of genetic testing services. This agent is involved in obtaining consent, facilitating family sharing opportunities, and providing a return of results [18]. The acceptability of this approach in health care systems has been shown through qualitative studies [16-18]. In other words, through individual interviews and focus groups, users were asked about their experience using the personalized conversational agent and whether they found it an acceptable alternative to talking with an actual genetic counselor in regard to obtaining consent, cascade testing, and returning of results.

### Objective

Despite the increasing use of conversational agents clinically and their initial acceptance, studies have yet to examine how patients interact with conversational agents implemented within a health care system to deliver cancer genetic services. To address this important research need, we will report on user interactions with a conversational agent in the delivery of pretest genetics education in a feasibility study through descriptive analysis of the information selected, time spent to complete the chat, and use of the open-ended question feature.

## Methods

### Participants

For this study, our team used a standards-based clinical decision support infrastructure to identify primary care patients without cancer in the University of Utah’s health care system who were eligible for cancer genetic evaluation. This algorithm used cancer family history data available in the electronic health record (EHR) to identify those who met the National Comprehensive Cancer Network guidelines for genetic testing for hereditary breast or ovarian [33] and colorectal cancer based on their family history [34]. All identified patients in this study were English-speaking, between the ages of 25 and 60 years, had a primary care appointment in the past 3 years, had no prior cancer diagnosis other than nonmelanoma skin cancer, had no prior genetic counseling or testing related to hereditary cancer, and had a patient portal account.

### Study Procedures

**Figure 1:**
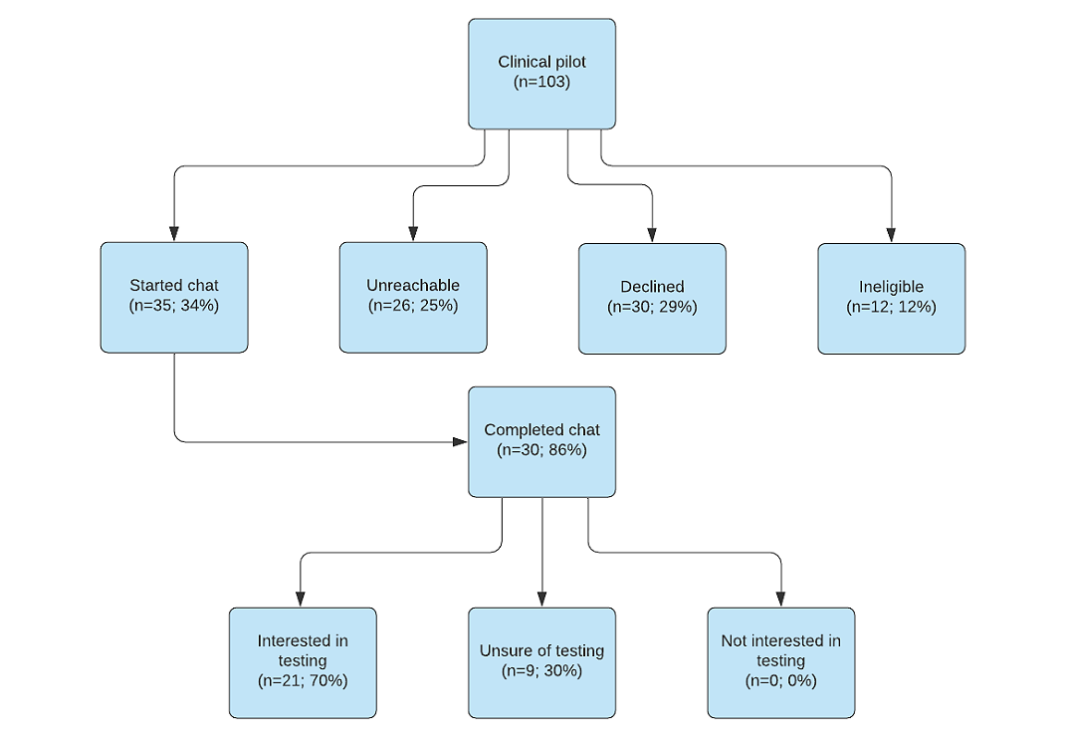
Study flow diagram.

From February to June 2020, a sample of 103 identified patients received a message through the patient portal about their eligibility for genetic services and an invitation to complete a pretest genetics education chat with the conversational agent (Figure 1). Of the 103 patients, 12 (11.7%) were ineligible for the study (because of relocation, previous testing, or incomplete family history) as determined through an EHR review and follow-up communication with the patient. The patients who did not complete the chat received a second patient portal message and up to three follow-up telephone calls to encourage engagement. Once the chat was completed, patients were contacted by a genetic counseling assistant who could answer questions and facilitate genetic testing for those who opted to test. A transcript of the pretest genetics education chat was added to the patient’s EHR and used for the analysis of interactions in this study. The study was approved by the University of Utah institutional review board.

### Content of Pretest Genetics Education Chat

The chat was developed by using the Health Insurance Portability and Accountability Act compliant with the Invitae platform. The content of the chat was based on the content of pretest genetic counseling delivered by certified genetic counselors at the Huntsman Cancer Institute, University of Utah, and the Perlmutter Cancer Center, New York University (Multimedia Appendix 1). Pretest genetic counseling provides information related to the purposes of genetic testing, genetic testing options, and the possible results generated [34-36]. Using an iterative design process, our interdisciplinary team, which comprised experts in genetic counseling, health communication, primary care, and cancer clinical genetics, scripted the chat content based on recordings of genetic counseling visits and clinical experience of the genetic counselors. Two rounds of both qualitative and quantitative user testing were completed to refine the content before the feasibility study described here.

The scripted pretest genetics education chat covered the major content areas in the following order: heritability of the risk for cancer, cancer risks associated with a mutation, genetic testing process, description of the types of genetic tests, and possible genetic testing outcomes and their implications. At the end of the chat with the conversational agent, patients were asked whether they were interested in continuing with the testing (yes, no, or unsure).

**Figure 2:**
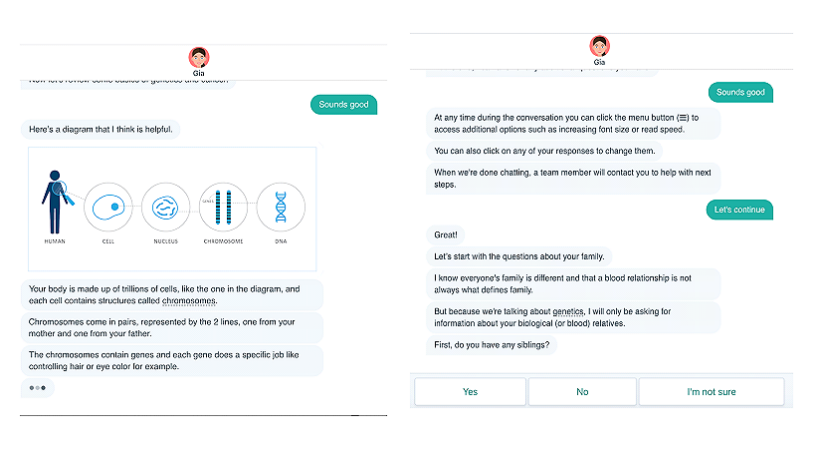
Genetic information assistant screenshots.

When patients opened the chat, the conversational agent first oriented them to the response buttons, menu (eg, to change text size and speed), and purpose of the chat. In addition, there was an introductory video filmed with the lead genetic counselor at the Huntsman Cancer Institute explaining the purpose of the chat and the conversational agent in the context of cancer genetic testing to add a human face and gain credibility with the user. The conversational agent then provided the scripted information in a real-time message format, with text bubbles containing 3 periods (ellipsis) to mirror an instant message conversation (Figure 2). All patients saw a core set of content decided upon by the team as essential for pretest genetics education. To supplement this core content at predetermined points throughout the conversation, options for responses were given on the bottom of the screen, and the patient’s response determined the next content delivered. Additional information included a range of topics, such as asking for basic explanations of cancer and genetics, the various possible results of genetic testing, and genetic mutation risk for various cancer types (eg, breast, pancreatic, and colon). This process allowed patients to choose to receive more or less details on a key topic (eg, goal, benefit, and risk) based on their preferences.

In addition, throughout the chat, participants were able to ask free-text questions. Natural language processing was used to answer the questions, if possible, from a library of prescripted responses. This created a real-time communication experience with immediately answered inquires that did not require additional effort from a provider for a response. If the platform’s artificial intelligence (AI) library was unable to match an answer to the patient’s open-ended question, they were prompted with alternative questions related to the topic of interest that had prescripted answers assigned. If the system was not able to determine an appropriate response to a question, it was sent via email to the clinical care team.

### Analysis

From the EHR, we abstracted data on patients’ sex, age, family history of cancer, race, and ethnicity. To analyze how patients interacted with the conversational agent, we collected the following information from the chat transcripts upon completion of the chat: time spent interacting with the conversational agent (with the exclusion of 2 cases in which users left the chat idle for >12 hours on their browser, for which we were unable to ascertain full time in interaction), options selected within the chat for supplemental content, and the number and types of open-ended questions asked by patients. A total of 2 coders independently extracted transcript data for 20% of the transcripts and had 94%-100% agreement for each code. Discrepancies were resolved by the full research team. From the study transcript records, we abstracted whether patients decided to continue with genetic testing after the chat. Data analysis was completed using SPSS version 25 (IBM Corp). We conducted 2-tailed *t* tests to examine differences in continuous variables between those patients who were sure of pursuing genetic testing and those who were not sure, as well as in demographics. Statistical significance was determined as *P*<.05.

## Results

### Overview

We identified and sent outreach messages via the patient portal to 103 patients. Of those 103, 12 (11.7%) were ineligible for the study (because of relocation, previous testing, or incomplete family history) and thus were excluded from the rest of the analysis. Of the 91 eligible patients, 75 (82%) opened the patient portal message with the chat link. About half of those participants (36/75, 48%) clicked the link to start the chat. Of those who started the chat, most finished it (30/36, 83%). As shown in Table 1, most of the patients who completed the chat were female (23/30, 77%), White (28/30, 93%), non-Hispanic or Latino (27/30, 90%), and had a mean age of 43.3 years (SD 9.96 years). Most patients had family histories of breast, ovarian, and pancreatic cancers, with no significant differences in these characteristics between those who did and did not complete the chat.

**Table 1:** Demographics and family histories of all invited users (N=91).

Demographics		Completed chat (n=30)	Did not complete chat (n=61)
Age (years), mean (SD)		43.3 (10.0)	42.1 (10.2)
**Sex, n (%)**			
	Female	23 (77)	50 (82)
	Male	7 (23)	10 (16)
**Race, n (%)**			
	White	28 (93)	55 (90)
	Black	N/A^a^	1 (2)
	Asian	N/A	2 (3.2)
	Other or did not disclose	2 (7)	2 (3.2)
**Ethnicity, n (%)**			
	Non-Hispanic or Latino	27 (90)	55 (90)
	Hispanic or Latino	2 (7)	4 (6)
	Did not disclose	1 (3)	1 (2)
**Family history of cancer,^b^ n (%)**			
	Breast	10 (30)	20 (28)
	Ovarian	9 (27)	21 (30)
	Pancreatic	8 (24)	19 (27)
	Colon	4 (12)	6 (8)
	Prostate	1 (3)	4 (6)
	Stomach	1 (3)	1 (1)

^a^N/A: not applicable.

^b^Patients could have >1 family member with a history of cancer, so the percentage values are not mutually exclusive.

At the completion of the educational content, users were asked if they wished to proceed with testing. As shown in Table 2, most users who completed the chat wished to continue with testing (21/30, 70%). Of these 30 users, 9 (30%) were unsure, whereas none indicated that they did not want to test. Age did not differ across groups (44 vs 42 years; *P*=.66); however, total time spent on the chat (10-14.1 minutes; *P*=.03), requests for additional information (1.2-4 requests; *P*=.03), and open-ended questions (0.3-1 open-ended questions; *P*<.001) did differ between those who decided to test and those who were unsure. The range of total time interacting with the chat was 6-31 minutes; however, most users spent 15-20 minutes with the chat. Those who indicated that they wanted to test spent an average of 10 (SD 2.57) minutes on the chat, selected 1-2 (mean 1.87, SD 1.2) options requesting additional pieces of information, and generally did not ask an open-ended question (Table 2). Those who were unsure spent 4 more minutes on average (mean 14.1, SD 7.41) with the chat and selected 3-4 (mean 3.67, SD 2.9) options requesting additional information. Of the 9 unsure patients, 4 (44%) asked 2 open-ended questions, with 1 participant asking 3 open-ended questions and 2 asking no open-ended questions.

**Table 2:** Completed chat continuous measures (N=30).

Continuous measures	Decided to test (n=21), mean (SD; range)	Unsure about testing (n=9), mean (SD; range)	All, mean (SD; range)
Age (years)	43.81 (9.15; 30-59)	42.0 (12.14; 25-60)	43.27 (9.96; 25-60)
Time spent on chat (minutes)^a,b^	10.0 (2.57; 6-15)	14.1 (7.41; 9-31)	11.17 (4.71; 6-31)
Total additional information items asked (GIA^c^ initiated)^b^	1.87 (1.24; 0-5)	3.67 (2.92; 1-9)	2.4 (2.0; 0-9)
Total open-ended questions asked^b^	0.095 (0.30; 0-1)	1.11 (1.05; 0-3)	0.4 (0.77; 0-3)

^a^N=28; 2 cases were excluded because the total time spent on chat was not collected.

^b^*P*<.05 (exact *P* values reported in main text).

^c^GIA: genetic information assistant.

In examining patients’ selections of options to request more information, there was a mean of 2.4 requests (SD 2.0; median 2.0, range 0-9) across all users. Only 1 patient asked for no additional pieces of information. As shown in Table 3, the most common topics for which patients requested more information were basic information about genetics and cancer (28/30, 93%), what types of risk factors were used to assess their risk (9/30, 30%), the genes that were included in the genetic test (9/30, 30%), and what options exist to lower cancer risk (9/30, 30%). Of the 10 patients who requested three or more additional pieces of information, all requested more information on basic genetics and cancer, 7 (70%) wanted more information on what genes were included in the test, and 8 (80%) wanted to know what options exist to mitigate cancer risk, whereas the rest of the selections varied.

**Table 3:** Use of options to request additional information (N=30).

Additional information requested		Value, n (%)
Basic information about cancer and genetics		28 (93)
Definition of sporadic causes of cancer		0 (0)
**Increase in cancer risk related to a positive genetic test result**		1 (3)
	Reason for range of cancer risk estimates	0 (0)
	Risk estimates for other cancers	0 (0)
How patients were identified		9 (30)
What genes are included on the test		9 (30)
**Options for decreasing cancer risk**		9 (30)
	Surgical or medical options for decreasing cancer risk	1 (3)
**Possible types of results from genetic testing**		
	Positive	3 (10)
	Negative	2 (7)
	Variant of unknown significance	3 (10)
Privacy protections		1 (3)
Any additional open-ended questions?		8 (27)
**Participant initial decision**		
	Continue with testing	21 (70)
	Unsure about continuing with testing	9 (30)

### Open-ended Questions

About one-quarter of the patients (8/30, 27%) typed in an open-ended question; however, a total of 11 questions were posed to our AI-based preprogrammed library (Textbox 1). Of the 9 users, 5 (56%) asked 1 question, 3 (33%) asked 2 questions, and 1 (11%) asked 3 questions. Open-ended questions often related to the cost of genetic testing with insurance and how this testing was different from direct-to-consumer genetic testing panels. Other question topics included how long testing results would take and whether health conditions could interfere with genetic testing results. Of the total 11 open-ended questions, 3 (27%) were directed to the clinical care team for follow-up and clarification; of these 3 questions, 1 (33%) was worded as a request rather than a question (“Let me know if I need to pay $0 out of pocket”). One open-ended question was clarified using the platform’s AI library by providing alternate questions that related to the proposed open-ended question, with the patient selecting the closest one, whereas the remaining questions could be answered directly through scripted responses in the library.

**List of open-ended questions asked by patients.**

**Open-ended questions**

- “Do you look at Aunts, Uncles, Cousins?”
- “Please let me know if my genetic testing would be greater than $0 out of pocket” (the questions were sent to the clinical care team).
- “How will this work with my insurance?”
- “Are there any health conditions that can interfere with the accuracy of the genetic test results?”
- “How much does the test cost?”
- “How long does it take to get test results?”
- “Is this different than 23andme and Ancestry?”
- “Why should I get screened?”
- “Will Healthy U Medcaid cover the cost of the test?”
- “How will this work with my insurance?”
- “What if I already have done the ‘Color’ genetic testing?”

## Discussion

### Principal Findings

Although conversational agents are increasingly important in precision medicine and are a potential alternative to educational sessions conducted by a person [11], there is a need to understand how users utilize and interact with the conversational agent to know what components (eg, topics and interface) may be most important to them. These data can add to prior findings on the acceptability and usability of conversational agents in multiple different contexts (behavior change, mental health, adherence, and genomics) [8-14,16-18]. Understanding what components are most salient and how users interact with the platform will enable providers, designers, and researchers to improve engagement with conversational agents for better patient experiences and potentially improved health outcomes. To our knowledge, this is the only study that has characterized the use of a conversational agent in a genomic setting, adding to the literature assessing its acceptability, usability, and understanding in various health contexts. Our findings suggest that the automated conversational agent approach engaged about one-third of the eligible patient population in clinical cancer genetic testing, with moderate outreach attempts and no health care appointments, highlighting potential scalability in its broader use as a potential cost-saving measure as well.

Although not directly asked of study participants, our conversational agent generally met the information needs of patients considering cancer genetic testing based on the limited use of open-ended questions. Patients asked for 2.4 additional pieces of information on average. More specifically, in addition to the core set of information that all patients received, patients mostly wanted more information on the basics of genetics and cancer, what types of risk factors were used to assess their risk, what genes were included in the genetic test, and what options exist to lower cancer risk. Additional information sought via open-ended questions related to cost, differences from direct-to-consumer genetic testing, whether health conditions could interfere with results, and how long results would take. A subset of patients spent more time on the chat, asked for additional pieces of information, asked more open-ended questions, and were unsure about testing at the end of the chat. Such high information–seeking patients may need additional support from a clinical provider to make a testing decision. However, the use of a conversational agent may substantially reduce provider burden by meeting the educational needs of most patients. Genetic counseling team members can then follow up with patients who have additional questions and concerns.

Our results are consistent with a previous study completed in the same patient population pool using the clinical decision support algorithm and standard of care approach involving a scheduled genetic counseling appointment [35]. Regardless of the delivery approach, both approaches (our automated conversational agent and the standard of care) reached just over a 30% unsolicited outreach uptake rate for clinical cancer genetic testing. It is important to note that testing use uptake did not differ by gender, age, and family history of cancer. However, it is important to consider other variables in the future, such as eHealth literacy and attitudes and beliefs related to cancer genetic testing uptake, as each approach may work differently and be most appropriate for different populations and within other contexts.

The findings further showed that when patients wanted additional information, they often selected one of the presented options rather than creating their own open-ended question, which highlights the importance for researchers, health care providers, and communication specialists to carefully design these options. Future research in genome-specific conversational agent contexts should examine why patients do and do not enter open-ended questions and whether there is a way to further improve chat interactivity via this feature. Our findings suggest that interactivity, in our case users interacting with our automated conversational agent, may be an important part of informing users regarding a particular topic, as suggested by the cognitive theory of multimedia learning [36] and adult learning theory [37]. Drawing more from these theories enables our understanding of how to leverage interactivity to encourage the elaboration of genetic testing technology while assessing knowledge increases. The development of conversational agents has underutilized theory and theory-driven concepts [8,38], such as interactivity. The greater use of theory may lead to more effective educational efforts as well as findings that are more generalizable across contexts, informing evidence-based strategies on how to best engage with users through interactions with conversational agents.

Our study expands upon a prior study that used focus groups to assess the acceptability, usability, and understanding of conversational agents for consent, follow-up, and cascade genomic testing [18]. The prior study found that users strongly supported the use of conversational agents in the context of providing genomic services. Although this prior study found that the use of the AI library of responses to open-ended questions was very appealing to users [18], in our study, we found that users made limited use of open-ended questions in actual practice. This could have also been because of the amount of predetermined information content in the conversational agent’s script.

In one other use of a conversational agent for a related application in the context of chronic conditions (eg, cardiovascular disease, diabetes, and cancer), Wang et al [39] developed an animated virtual counselor to collect electronic family health histories for clinical risk assessment [29,30] and as a proxy for genetic predisposition to personalize medical care and disease prevention [40-45]. In a randomized comparison with the Surgeon General’s My Family Health Portrait [46], the conversational agent had better acceptability and usability outcomes (eg, ease of use, flow, understanding information, and satisfaction). However, the study did not assess in detail how users interacted with the virtual counselor for knowing your family history. Therefore, we enhance the previous understanding of both the acceptability and usability of conversational agents and how their features are used in actual practice.

### Strengths and Limitations

As our study was a feasibility study, it had some limitations. One of our limitations was the composition of our cohort, which primarily comprised White, older women. The use of conversational agents in other populations may differ. We restricted our study to patients aged 25-60 years because screening and prevention recommendations can be modified for those in this range with inherited cancer susceptibility or familial risk [47-49]. Our cohort being predominantly middle-aged may have affected the level of engagement we saw with the conversational agent. Second, we were not able to examine how long a patient actually interacted with the conversational agent as opposed to merely having the chat open. It is possible that users left the chat idle and came back to it at a later time. Eye-tracking and other laboratory-based studies will allow further examination of these issues. Finally, we did not directly ask the users if their informational needs were met, which is an important next step. However, with the lack of open-ended questions being asked by users, we believe that the conversational agent was able to effectively facilitate decision-making for cancer genetic testing. More research on user experience and participant perceptions of informational needs is needed in this area.

### Conclusions

Despite these limitations, our study’s results suggest that a conversational agent can meet the information needs of primary care patients and can represent a scalable alternative for pretest counseling for patients considering cancer genetic testing. With the increased demand for genetic testing and counseling, through the development, implementation, and maintenance of conversational agents, such as the one presented in our study, this strategy has the potential to save operating costs and improve the availability of these technologies for underserved groups. This indicates that our conversational agent may be an acceptable alternative (or supplement) to an in-person genetic counseling pretest visit, although outcomes such as use of testing should be evaluated in a randomized trial. In addition, we learned how patients interact with our conversational agent, what additional information is of most interest, and the patients’ interest in using the open-ended question feature. We also found that patients who were unsure about testing tended to ask for more information, asked open-ended questions, spent more time with the chat, and may need additional interpersonal support and information for decision making.

## Appendix

Multimedia Appendix 1 Genetic information assistant chat script for pretest educational conversation.

## Abbreviations

AI: artificial intelligence
EHR: electronic health record
