# Supplementary material for: Patient Interactions With an Automated Conversational Agent Delivering Pretest Genetics Education: Descriptive Study
Source: J Med Internet Res. 2021 Nov 18;23(11):e29447. doi: 10.2196/29447 (PMC8663668; doi:10.2196/29447)
Supplement: Multimedia Appendix 1 [file jmir_v23i11e29447_app1.docx]

| **Patient receives this pretest education chat if identified by BRIDGE as meeting criteria for testing.** | | | | | |
| --- | --- | --- | --- | --- | --- |
| Gia: Hi, {patientName}.  Gia: I’m GIA, your automated Genetic Information Assistant.  Gia: On behalf of the University of Utah healthcare team, I’m reaching out to patients who might benefit from genetic testing.  Patient: Hi Gia  Gia: Before we get started, here’s a quick message from Wendy at University of Utah genetics.  *** INTRO VIDEO ***  Patient: Let’s continue  Gia: Based on the family health history in your medical record, you meet criteria for testing of genes related to certain cancers.  Gia: The healthcare team at University of Utah recommends having this genetic testing to provide more information about your cancer risk and to help plan your care.  Gia: It can also be important for your family members, including children if you have them.  Patient: I see  Gia: In this chat I’ll ask you some basic information about your family, and provide information about why testing is important, how it is done, and what results are possible.  Gia: At the end, I can answer any additional questions you have.  Gia: At any time during our conversation you can click the menu button (☰) to access additional options such as increasing font size and read speed.  Gia: You can also click on any of your responses to change them.  Gia: When we're done chatting, you'll be able to access a copy of our conversation in your MyChart account. | | | | | |
| Patient: Let’s continue | Patient: I’ll come back later  Gia: Ok, when you come back we’ll continue where we left off.  Gia: The sooner you complete our chat, the sooner we can take next steps.  Patient: I’m ready to continue now | | | | |
| Gia: Great!  Gia: Let’s start with the questions about your family.  Gia: I know everyone's family is different and that a blood relationship is not always what defines family.  Gia: But because we're talking about genetics, I will only be asking for information about your biological (or blood) relatives.  Gia: First, do you have any siblings?  Patient: Yes / No / I’m not sure  Gia: How many brothers do you have? (includes full and half brothers)  Patient: *Types in*  Gia: How many sisters do you have? (includes full and half sisters)  Patient: *Types in*  Gia: And do you have any children?  Patient: Yes / No  Gia: How many sons do you have?  Patient: Chooses from options 0 - 10  Gia: How many daughters do you have?  Patient: Chooses from options 0 - 10  Gia: Thank you for answering those questions.  Gia: Now let’s review some basics of genetics and cancer. | | | | | |
| Patient: Sounds good  Gia: Here’s a diagram that I think is helpful.  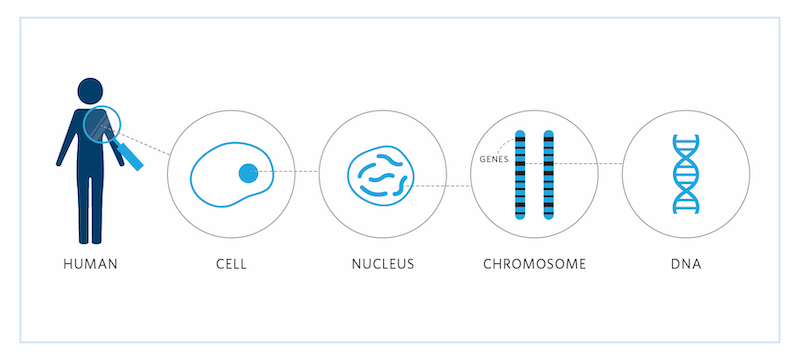  Gia: Your body is made up of trillions of cells, like the one in the diagram and each cell contains structures called chromosomes.  Gia: Chromosomes come in pairs, represented by the 2 lines, one from your mother and one from your father.  Gia: The chromosomes contain genes and each gene does a specific job such as controlling hair or eye color.  Gia: Sometimes a gene has a change in the genetic code. A change that keeps a gene from doing its job correctly is called a mutation.  Patient: I see / This is sounding familiar now  Gia: Some genes have the job of preventing cancer.  Gia: If a person has a mutation in a gene that is supposed to be preventing cancer, then their risk of cancer can be increased.  Gia: It doesn’t mean that a person will definitely get cancer and their risk depends on many factors.  Patient: Ok / Interesting! | | | Patient: I have a good understanding of genetics  Gia: Great. As you may know, your cells contain chromosomes which come in pairs, one from your mother and one from your father.  Gia: The chromosomes contain genes and each gene does a specific job such as controlling hair or eye color.  Gia: Sometimes a gene has a change in the genetic code. A change that keeps a gene from doing its job correctly is called a mutation.  Gia: If a person has a mutation in a gene whose job is to prevent cancer then their risk of cancer can be increased.  Gia: It doesn’t mean that a person will definitely get cancer and their risk depends on many factors.  Patient: Ok / Yes, we’re on the same page | | |
| Gia: Cancer can be inherited, or it can occur randomly.  Gia: Cancer that is caused by a gene mutation is “hereditary”, meaning that it is passed down within families.  Gia: Other cancer is random, or “sporadic,” which means that it’s caused by things like aging, lifestyle, and environment. | | | | | |
| Patient: I understand | | | Patient: Can you explain this in another way?  Gia: Yes! Here's a short video that explains it nicely.  *** Ambry Umbrella Video ***  Patient: Thanks, that was helpful / Let’s continue | | |
| Gia: Everyone has some level of cancer risk. Most people have what is known as "average" or "population" risk.  Gia: People with a close family member with cancer, such as a parent or sibling, generally have an increased risk for developing that type of cancer.  Gia: Those that test positive for a gene mutation related to cancer may have an even higher risk. | | | | | |
| Patient: I see | Patient: Can you give me an example?  Gia: Of course!  Gia: Here's an illustration of lifetime breast cancer risk for a woman. ***IMAGE***  Gia: The average lifetime risk of breast cancer is 10%.  Gia: Having a mother or sister with breast cancer increases the risk to 15 - 25%.  Gia: And those with gene mutations in the gene called *BRCA1* have a 50 - 70% risk for breast cancer. | | | | |
|  | Patient: Thanks, that helps | Patient: Why is there a range of percentages?  Gia: The exact risk depends on many factors including the specific gene mutation, if there is one, and the details of the family history.  Gia: You can talk to a genetic counselor to help you understand your personal risk. | | | Patient: Can I see more examples?  *Loop through offering other examples below* |
|  |  | Patient: Ok | | Patient: Can I see more examples?  *Loop through offering other examples below* |  |

Ovarian cancer

| Gia: Here's an illustration of lifetime ovarian cancer risk for a woman. ***IMAGE***  Gia: The average lifetime risk of ovarian cancer is 1%.  Gia: Those with family history have a risk of 3 - 5%.  Gia: And those with a high-risk gene mutation have a risk of 20 - 50%. |
| --- |

Colon cancer

| Gia: Here's an illustration of lifetime colon cancer risk. ***IMAGE***  Gia: The average lifetime risk of colon cancer is 5%.  Gia: Those with family history have a risk of 8 - 10%.  Gia: And those with a high-risk gene mutation have a risk of 20 - 50%. |
| --- |

Pancreatic cancer

| Gia: Here's an illustration of lifetime pancreatic cancer risk. ***IMAGE***  Gia: The average lifetime risk of pancreatic cancer is 1%.  Gia: Those with family history have a risk of 2 - 3%.  Gia: And those with a high-risk gene mutation have a risk of 5 - 20%. |
| --- |

| Gia: There are specific things we look for in a family’s cancer history to figure out whether the cancer is likely to be hereditary.  Gia: You may have been identified as eligible for genetic testing because we found one or more of these things in your medical record. | | |
| --- | --- | --- |
| Patient: What kind of things do you look for?  Gia: Some of the things we look for are:  Gia: Having cancer at a young age (usually younger than 50)  Gia: Having multiple family members with cancers that are known to share a common genetic link  Gia: Some rare cancer types such as ovarian cancer, men with breast cancer, or pancreatic cancer  Gia: Jewish ancestry in combination with certain cancers  Patient: Got it | | Patient: Ok, Gia |
| Gia: The genetic testing we are offering you looks at many genes that are related to hereditary cancer.  Gia: Each gene is unique in terms of which cancer(s) it relates to, the level of risk it causes, and how it impacts care.  Gia: Every gene we test for is associated with cancers that have screening and prevention options. | | |
| Patient: Ok | | Patient: Which genes are included?  Gia: We test for mutations in genes you may have heard of like *BRCA1* and *BRCA2*.  Gia: And also, for many other genes that you likely are not familiar with.  Gia: If you have questions about specific genes, we can answer those at the end of the chat.  Patient: Got it |
| Gia: If we find a mutation in one of these genes, additional or more frequent cancer screening may be recommended to help detect cancer early.  Gia: For some types of cancer there might be other options that can help lower cancer risk.  Patient: Ok / What kind of options? / Do I have to get testing? | | |
| Patient: Do I have to get tested?  Gia: Testing is your choice, but this information could help your healthcare team plan what cancer screening is best for you.  Gia: If you decide to get tested, you will be well supported.  Gia: If you decide not to get genetic testing, we can only give recommendations for cancer screening based on your personal and family history.  Gia: At the end of this chat you can decide if you want testing and can talk to a genetics professional to get more information.  Patient: Ok | Patient: What kind of options?  Gia: It depends on your specific situation.  Gia: For example, if you are at high risk of developing breast cancer, you may be offered a mammogram and breast MRI every year to screen you more closely.  Gia: Surgical options or medications may be considered with some mutations.  Patient: Ok / Do I have to have testing? | |
| Gia: There are 3 types of results that we may find when we look at your genes. Let me describe them for you:   1. **Positive**: A gene mutation was found that increases the chances of developing certain cancers. Laboratories may call a mutation a “pathogenic variant” on a test report. 2. **Negative**: No gene mutations that increase the chance of developing cancer were found in any of the genes tested. 3. **Variant of Unknown Significance (VUS)**: A variation was found in one of your genes, but the variation has not been proven to increase the risk of cancer. This is not a positive result and should not be used to change your medical management. | | |

| Patient: More on positive result  Gia: If you have a positive result, your doctor or a genetic counselor will walk you through the next steps.  Gia: It’s important to know that if you test positive, it does not mean you will definitely develop cancer. | | Patient: More on negative result  Gia: A negative result would help reassure you that you do not have a gene mutation causing a higher risk for cancer.  Gia: However, you may still have an increased risk for cancer based on your family history.  Gia: A genetic counselor will review your family history and let you know what cancer screening is recommended.  Gia: Genetic testing of a family member who had cancer may provide more information about whether there is a gene mutation in your family.  Gia: The genetic counseling team can help a relative have genetic testing. | Patient: More on VUS result  Gia: All people have variations in their genes. This is part of what makes each person unique.  Gia: Sometimes genetic testing identifies variations and we can’t say for sure yet if they are linked to cancer.  Gia: As we learn more, most of these variants are found to be normal (not associated with cancer or other disease).  Gia: If we find you have a VUS we will keep track of it and notify you when we learn more about it.  Patient: Got it / 👍  Gia: Even with a VUS result, you may still have an increased risk for cancer based on your family history.  Gia: A genetic counselor will review your family history and let you know what cancer screening is recommended. | Patient: Let’s move on  Gia: Sounds good  Gia: I just want to make sure you know that if you test positive, it does not mean you will definitely develop cancer. |
| --- | --- | --- | --- | --- |
| Patient: Let’s move on / Ok | Patient: Let’s move on / Ok  *If didn’t already cover more info on positive result (otherwise skip this section)*  Gia: Sounds good  Gia: If you have a positive result, your doctor or a genetic counselor will walk you through the next steps.  Gia: It’s important to know that if you test positive, it does not mean you will definitely develop cancer.  Patient: Ok | | | |
| Gia: If you are found to have a mutation, it will be important to share this information with family members.  Gia: Close relatives like children, siblings and parents have a 1 in 2 (or 50%) chance of having the same mutation and other relatives may also have increased risk.  Gia: If needed, we can help your relatives get testing. | | | | |
| Patient: Got it | Patient: How is privacy protected?  Gia: Only you and your healthcare providers will have access to your results.  Gia: Your report will go into your medical record so those involved in your care can review it as needed.  Gia: It is up to you whether you choose to share your genetic test results with others outside of the healthcare system.  Gia: However, as we have discussed it is often very helpful to share your genetic test results with family members.  Patient: Ok  Gia: Also, you are protected by GINA, the Genetic Information Nondiscrimination Act.  Gia: GINA provides specific protections related to health insurance and employment. For more complete information click **here**.  Patient: Ok / Thank you | | | |
| Gia: If you’re interested in genetic testing for cancer risk, the genetics team will give you a call to discuss next steps.  Gia: We’ll work with you on insurance coverage. Out of pocket cost is usually $100 or less.  Gia: Before I ask if you're interested in testing, do you have any additional questions?  **Clear AI**  Gia: What do you think? Would you like to move forward? | | | | |
| Patient: Yes  Gia: Great. When we call you, we’ll go over instructions for providing a sample for the testing and answer any additional questions.  Gia: A kit can be sent to your home in the next 72 hours, and results will be available in 3 to 4 weeks after you send the sample. | | Patient: No  Gia: Ok. Someone on the team might reach out to you to make sure you got all the information you need. | | Patient: I’m not sure yet  Gia: Ok, I’ll pass that information on to the team.  Gia: Someone will reach out to you about your questions. |
|  |  |  |  |  |
| Gia: I'm putting a copy of our conversation in your MyChart account.  Gia: Have a great day. Goodbye!  *end chat* | | | | |

Hover-over verbiage (updated)

Mutation: A change in the genetic code that increases cancer risk

Hereditary: Passed down within families

Positive: A gene mutation was found that increases the chances of developing certain cancers

Cancer screening: Looking for cancer at an early stage before a person has any symptoms

Gene: A gene is the basic unit of heredity. Genes can have mutations that affect cancer risk.

Genetic code: A person’s complete set of genes

Genetic testing: A type of medical test that looks for changes in genes

Chromosome: DNA is organized in chromosomes. Genes are found on chromosomes.

Medical record: A record of a patient's medical information, for example, disease conditions, test results, care received, and medicines taken.
